# Diversities of disability caused by lung cancer in the 66 Belt and Road initiative countries: a secondary analysis from the Global Burden of Disease Study 2019

**DOI:** 10.3389/fonc.2023.1247006

**Published:** 2023-11-10

**Authors:** Zhenfeng Zhu, Wenjing Ye, Li Zhang, Wenchang Jia, Binghong Chen, Qizhe Wang, Xuelin Cheng, Shijia Yang, Zhaoyu Zhang, Yibo Ding, Xiaopan Li

**Affiliations:** ^1^ Department of Integrative Medicine, Zhongshan Hospital, Fudan University, Shanghai, China; ^2^ Department of Respiratory Medicine, Xinhua Hospital, School of Medicine, Shanghai Jiao Tong University, Shanghai, China; ^3^ Department of Cancer Prevention, Centers for Disease Control and Prevention, Shanghai, China; ^4^ School of Public Health, Fudan University, Shanghai, China; ^5^ Department of Health Management Center, Zhongshan Hospital, Fudan University, Shanghai, China; ^6^ Department of Epidemiology, Naval Medical University, Shanghai, China

**Keywords:** “B&R” countries, lung cancer, burden of disease, risk factors, average annual percent change, years lived with disability (YLDs), disability-adjusted life years (DALYs)

## Abstract

**Objectives:**

Due to the increase in life expectancy and the aging of the global population, the “Belt and Road” (“B&R”) countries are faced with varying degrees of lung cancer threat. The purpose of this study is to analyze the differences in the burden and trend of lung cancer disability in the “B&R” countries from 1990 to 2019 so as to provide an analytical strategic basis to build a healthy “B&R”.

**Methods:**

Data were derived from the Global Burden of Disease 2019 (GBD 2019). Incidence, mortality, prevalence, the years lived with disability (YLDs), and disability-adjusted life years (DALYs) of lung cancer and those attributable to different risk factors were measured from 1990 to 2019. Trends of disease burden were estimated by using the average annual percent change (AAPC), and the 95% uncertainty interval (UI) was reported.

**Results:**

China, India, and the Russian Federation were the three countries with the highest burden of lung cancer in 2019. From 1990 to 2019, the AAPC of incidence, prevalence, mortality, and DALYs generally showed a downward trend in Central Asia (except Georgia) and Eastern Europe, while in China, South Asia (except Bangladesh), most countries in North Africa, and the Middle East, the trend was mainly upward. The AAPC of age-standardized incidence was 1.33% (1.15%–1.50%); the AAPC of prevalence, mortality, and DALYs from lung cancer in China increased by 24% (2.10%–2.38%), 0.94% (0.74%–1.14%), and 0.42% (0.25%–0.59%), respectively. A downward trend of the AAPC values of age-standardized YLD rate in men was shown in the vast majority of “B&R” countries, but for women, most countries had an upward trend. For adults aged 75 years or older, the age-standardized YLD rate showed an increasing trend in most of the “B&R” countries. Except for the DALY rate of lung cancer attributable to metabolic risks, a downward trend of the DALY rate attributable to all risk factors, behavioral risks, and environmental/occupational risks was shown in the vast majority of “B&R” countries.

**Conclusion:**

The burden of lung cancer in “B&R” countries varied significantly between regions, genders, and risk factors. Strengthening health cooperation among the “B&R” countries will help to jointly build a community with a shared future for mankind.

## Introduction

The “Belt and Road” (“B&R”) Initiative refers to the “Silk Road Economic Belt” and the “21st Century Maritime Silk Road”, which was first proposed by China in 2013. “B&R” countries run through Eurasia, connecting the Asia Pacific Economic Circle in the east and the European Economic Circle in the west (1). “B&R” Initiative can fully rely on the existing bilateral and multilateral mechanisms between China and relevant countries and leverage existing and effective regional cooperation platforms. Health crises are cross-border issues that require collective action to address (2, 3). In 2017, the Chinese government proposed the “Health Silk Road” (HSR) initiative to strengthen global health cooperation. “B&R” health exchange and cooperation helps to share successful experiences in the medical and health field. HSR initiative can promote cooperation in health, build a strong and resilient health system for transnational cooperation, and jointly build a “community of human health” in order to deal with disease epidemics.

Lung cancer is one of the main causes of new cancer cases and cancer-related deaths worldwide (4). In the past two decades, significant improvements have been made in understanding the biology and targeted therapy in lung cancer and the application of immune checkpoint inhibitors (ICIs), which have changed the prognosis of many patients (5). In terms of disability-adjusted life years (DALYs), the disease burden is evolving to be dominated by the years lived with disability (YLDs) (6). YLDs measure the amount of time that people lose to illnesses and injuries that do not cause death but reduce health. These areas are becoming hot topics for measuring and improving health outcomes due to transitions in aging populations and mortality in different countries.

Currently, “B&R” member countries are facing varying degrees of lung cancer threat. It is crucial to have comparable and comprehensive analysis and assessment of lung cancer incidence, mortality, disease burden, and long-term trends in China and its partner countries in order to improve public health and the success of the organizations. However, little is known about the status and extent of lung cancer in the 66 countries under the “B&R” Initiative. Our objective is to estimate the burden and trends of lung cancer from 1990 to 2019 through this study, providing a basis for formulating disease prevention and control policies and building a “community of human health” by strengthening health industry cooperation among the “B&R” countries.

## Methods

### Data sources

This study was conducted using the Global Burden of Disease 2019 (GBD 2019) study obtained from the Institute for Health Metrics and Evaluation (IHME) website. All data for this study were obtained from the Institute for Health Metrics and Evaluation (IHME) website (https://www.healthdata.org/data-tools-practices/data-sources). Detailed methodology has been published elsewhere (7, 8).

### Estimation of lung cancer burden

Incidence, mortality, prevalence, YLDs, and DALYs were used in this study. Age-standardized rates for incidence, mortality, prevalence, YLDs, and DALYs were calculated according to a global age structure from 2019. YLDs were estimated by multiplying lung cancer prevalence with the corresponding disability weight. DALYs assess comprehensively premature death and the disease burden of disability. DALYs are equal to YLDs plus years of life lost (YLLs). YLLs are calculated as the product of counts of deaths caused by lung cancer and a standard remaining life expectancy at the age of death. The age-standardized rates were corrected by the direct method and the world standard population to account for differences in the population age structure. Our study follows the Guidelines for Accurate and Transparent Health Estimates Reporting (GATHER) to ensure transparency and replicability (**Table 1**) (9).

**Table 1:** GATHER checklist of information included in reports of global health estimates.

#	Checklist item	Section/paragraph/interpretation
Objectives and funding		
1	Define the indicators, populations, and time periods for which estimates were made.	Methods/”Data sources”
2	List the funding sources for the work.	Funding
Data inputs		
*For all data inputs from multiple sources that are synthesized as part of the study:*		
3	Describe how the data were identified and how the data were accessed.	As mentioned in the Methods/”Data sources” section, the details have been published previously.
4	Specify the inclusion and exclusion criteria. Identify all *ad-hoc* exclusions.	As mentioned in the Methods/”Data sources” section, the details have been published previously.
5	Provide information on all included data sources and their main characteristics. For each data source used, report reference information or contact name/institution, population represented data collection method, year(s) of data collection, sex and age range, diagnostic criteria or measurement method, and sample size, as relevant.	Available *via* online data source tools (http://ghdx.healthdata.org/gbd-2019/data-input-sources).
6	Identify and describe any categories of input data that have potentially important biases (e.g., based on characteristics listed in item 5).	As mentioned in the Methods, the details have been published previously.
*For data inputs that contribute to the analysis but were not synthesized as part of the study:*		
7	Describe and give sources for any other data inputs.	Available *via* online data source tools (http://ghdx.healthdata.org/gbd-2019/data-input-sources).
*For all data inputs:*		
8	Provide all data inputs in a file format from which data can be efficiently extracted (e.g., a spreadsheet as opposed to a PDF), including all relevant meta-data listed in item 5. For any data inputs that cannot be shared due to ethical or legal reasons, such as third-party ownership, provide a contact name or the name of the institution that retains the right to the data.	Available *via* online data source tools (http://ghdx.healthdata.org/gbd-2019/data-input-sources).
Data analysis		
9	Provide a conceptual overview of the data analysis method. A diagram may be helpful.	Flow diagrams of the overall methodological processes were available online (http://ghdx.healthdata.org/gbd-2019/code/nonfatal-12).
10	Provide a detailed description of all steps of the analysis, including mathematical formulae. This description should cover, as relevant, data cleaning, data pre-processing, data adjustments and weighting of data sources, and mathematical or statistical model(s).	As mentioned in the Methods/”Statistical analyses” section.
11	Describe how candidate models were evaluated and how the final models were selected.	As mentioned in the Methods/”Statistical analyses” section, the details have been published previously.
12	Provide the results of an evaluation of model performance, if done, as well as the results of any relevant sensitivity analysis.	As mentioned in the Methods/”Statistical analyses” section, the details have been published previously.
13	Describe methods for calculating uncertainty of the estimates. State which sources of uncertainty were, and were not, accounted for in the uncertainty analysis.	Methods/”Statistical analyses” section.
14	State how analytic or statistical source code used to generate estimates can be accessed.	Methods/”Statistical analyses” section.
Results and discussion		
15	Provide published estimates in a file format from which data can be efficiently extracted.	Results and online data tools (data visualization tools and data query tools, http://ghdx.healthdata.org/gbd-2019).
16	Report a quantitative measure of the uncertainty of the estimates (e.g., uncertainty intervals).	Results and online data tools (data visualization tools and data query tools, http://ghdx.healthdata.org/gbd-2019).
17	Interpret results in light of existing evidence. If updating a previous set of estimates, describe the reasons for changes in estimates.	Discussion, paragraphs 1–7
18	Discuss limitations of the estimates. Include a discussion of any modeling assumptions or data limitations that affect interpretation of the estimates.	Discussion, paragraph 8

GATHER, the Guidelines for Accurate and Transparent Health Estimates Reporting.

### “B&R” countries

**Figure 1:**
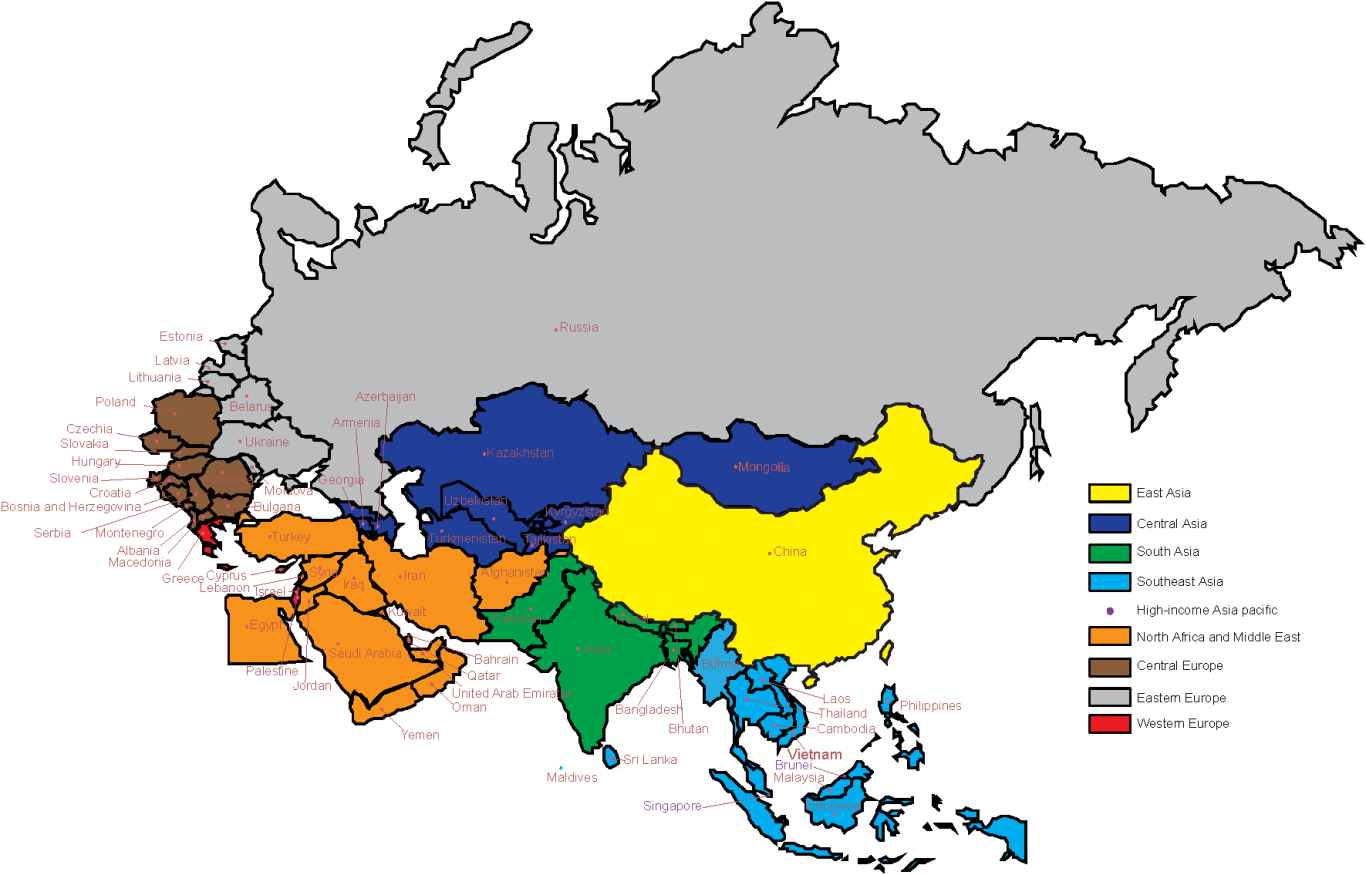
GBD regions of 66 B&R countries. GBD, Global Burden of Disease; B&R, Belt and Road.

The 66 members of “B&R” countries are as follows: 1) East Asia: China; 2) Central Asia: Armenia, Azerbaijan, Georgia, Kazakhstan, Kyrgyzstan, Mongolia, Tajikistan, Turkmenistan, and Uzbekistan; 3) South Asia: Bangladesh, Bhutan, India, Nepal, and Pakistan; 4) Southeast Asia: Cambodia, Indonesia, Laos, Malaysia, Maldives, Burma, the Philippines, Sri Lanka, Thailand, and Vietnam; 5) high-income Asia Pacific: Brunei and Singapore; 6) North Africa and the Middle East: Afghanistan, Bahrain, Egypt, Iran, Iraq, Jordan, Kuwait, Lebanon, Oman, Palestine, Qatar, Saudi Arabia, Syria, Turkey, the United Arab Emirates, and Yemen; 7) Central Europe: Albania, Bosnia and Herzegovina, Bulgaria, Croatia, Czechia, Hungary, Montenegro, North Macedonia, Poland, Romania, Serbia, Slovakia, and Slovenia; 8) Eastern Europe: Belarus, Estonia, Latvia, Lithuania, Republic of Moldova, Russia, and Ukraine; 9) Western Europe: Cyprus, Greece, and Israel. See **Figure 1** for more details.

### Statistical analyses

We calculated absolute numbers and age-standardized rates of incidence, mortality, YLDs, and DALYs to quantify the burden of lung cancer, grouped by gender and age in the “B&R” countries. Age-standardized estimates allow comparisons across time, countries, and subregions and are adjusted for differences in the age distribution of the population. Age was divided into three groups: 20–54 years, 55–74 years, and ≥75 years. The three risk factors (behavioral risks, environmental/occupational risks, and metabolic risks) were included in the present study. Data were stratified by region [high, high-middle, middle, low-middle, and low socio-demographic index (SDI)]. SDI is a composite indicator of a country’s lag-distributed income per capita, educational attainment, and the total fertility rate in women younger than 25 years. Methods of SDI development and computation are detailed elsewhere (10). Trends of disease burden from 1990 to 2019 were evaluated using average annual percent change (AAPC), which was calculated by the Joinpoint Regression Program (Version 4.9.0.0, March 2021) (11). Uncertainty intervals (UIs) of 95% were calculated with the 2.5th and 97.5th percentiles of 1,000 drawn by age, sex, location, and year (12). The map visualization of the “B&R” member states was performed using the “ggmap” package in R software (version 4.3.0, R Core Team). The “ggmap” package is an extension package, which obtains shapefiles from Google Maps. *p* < 0.05 was considered statistically significant.

### Patient and public involvement

Being involved in the Global Burden of Disease 2019 and other open databases rather than directly speaking to patients inspired this research. Although no patient was directly involved in this study, members of the public read our manuscript, and all agreed on the specific findings of this study.

## Results

### The absolute number of incidence, mortality, prevalence, YLDs, and DALYs due to lung cancer in 2019

The absolute number of incidence, mortality, YLDs, and DALYs in 2019 caused by lung cancer in each member country of the “B&R” are shown in **Table 2**. We noted that there were significant geographic differences in the number of lung cancer incidence, mortality, YLDs, and DALYs across countries, with China, India, and the Russian Federation being the three countries with the highest burden of lung cancer. In 2019, there were 832,922.16 (95% UI 700,293.15 to 981,631.63) lung cancer incidences, 757,171.25 (95% UI 638,741.18 to 887,751.81) deaths, 199,351.51 (95% UI 138,983.38 to 264,035.88) YLDs, and 17,128,584.02 (95% UI 14,340,490.76 to 20,231,342.32) DALYs due to lung cancer in China. The country with the lowest number of lung cancer incidences is the Maldives in Southeast Asia (26.49, 95% UI 21.86 to 31.61).

**Table 2:** The absolute number of incidence, mortality, prevalence, YLDs, and DALYs due to lung cancer in 2019.

Countries	Incidence		Mortality		Prevalence		YLDs		DALYs	
	Number	95% UI	Number	95% UI	Number	95% UI	Number	95% UI	Number	95% UI
East Asia										
China	832,922.16	700,293.15, 981,631.63	757,171.25	638,741.18, 887,751.81	1,137,880.03	950,548.16, 1,344,733.04	199,351.51	138,983.38, 264,035.88	17,128,584.02	14,340,490.76, 20,231,342.32
Central Asia										
Armenia	1,345.78	1,129.72, 1,595.96	1,333.76	1,121.61, 1,577.50	1,489.61	1,239.77, 1,771.27	306.99	214.18, 415.51	32,529.70	27,097.00, 38,700.25
Azerbaijan	2,375.07	1,746.37, 3,041.88	2,296.14	1,687.24, 2,920.62	2,746.29	2,010.70, 3,521.50	562.84	346.47, 816.76	67,384.39	49,095.71, 86,484.43
Georgia	1,783.98	1,487.37, 2,108.03	1,769.96	1,485.09, 2,079.83	1,948.70	1,613.94, 2,329.43	405.58	275.92, 552.45	45,743.10	37,924.68, 54,187.58
Kazakhstan	3,829.27	3,259.16, 4,405.84	3,716.64	3,184.40, 4,260.53	4,337.40	3,675.62, 5,018.90	886.63	608.48, 1,203.93	99,097.82	84,119.24, 114,546.55
Kyrgyzstan	568.77	492.88, 646.55	560.10	485.65, 635.73	636.25	550.11, 726.34	135.63	92.30, 187.42	15,377.13	13,279.03, 17,539.43
Mongolia	662.38	512.20, 863.46	671.76	522.79, 869.20	691.49	531.75, 911.41	150.83	97.87, 218.46	17,529.17	13,388.76, 23,175.99
Tajikistan	593.28	478.94, 740.28	585.82	476.34, 731.84	665.01	531.77, 838.13	143.33	93.00, 206.53	17,301.77	13,762.61, 21,771.91
Turkmenistan	412.75	326.10, 520.92	399.22	316.22, 503.72	478.24	376.27, 606.32	101.55	65.45, 147.24	11,915.03	9,357.08, 15,098.17
Uzbekistan	2,770.81	2,282.13, 3,324.29	2,656.35	2,188.76, 3,178.98	3,246.61	2,682.20, 3,914.21	672.72	444.33, 942.78	80,569.90	66,295.49, 96,840.99
South Asia										
Bangladesh	9,652.31	6,331.50, 15,119.70	9,970.50	6,568.78, 15,550.09	9,890.12	6,398.48, 15,551.36	2,280.53	1,252.41, 4,049.16	245,789.45	158,425.89, 385,218.27
Bhutan	42.59	30.65, 58.41	44.43	32.07, 60.68	43.39	31.15, 59.94	9.94	6.19, 15.08	1,071.70	764.16, 1,473.98
India	87,339.21	71,865.33, 103,504.12	89,241.82	73,674.82, 105,402.83	90,057.70	73,919.11, 106,986.69	20,367.52	14,189.78, 27,535.34	2,275,225.20	1,871,749.98, 2,691,295.16
Nepal	1,759.15	1,263.39, 2,275.02	1,837.31	1,333.96, 2,370.52	1,763.67	1,266.75, 2,294.01	412.71	253.56, 614.19	45,196.18	32,343.42, 59,231.11
Pakistan	18,401.25	13,969.72, 24,265.21	18,550.27	14,209.46, 23,969.10	19,631.92	14,826.19, 26,083.02	4,258.89	2,794.58, 6,129.06	522,647.78	400,317.47, 680,663.37
Southeast Asia										
Cambodia	2,887.60	2,266.60, 3,576.17	2,985.13	2,340.29, 3,698.58	2,961.87	2,290.27, 3,680.80	649.54	419.97, 907.18	76,026.47	58,766.84, 94,334.05
Indonesia	48,198.90	35,265.54, 59,309.34	49,437.42	36,066.08, 61,104.64	50,233.86	36,772.54, 62,097.63	10,918.72	6,937.99, 15,201.44	1,279,980.70	927,626.41, 1,596,039.79
Lao	973.36	717.55, 1,264.48	999.82	742.13, 1,290.39	1,007.06	730.37, 1,329.52	220.41	135.86, 318.98	26,517.75	19,142.26, 35,091.64
Malaysia	5,164.65	3,997.20, 6,560.78	5,221.07	4,059.90, 6,639.41	5,544.16	4,257.62, 7,066.08	1,176.14	742.18, 1,703.49	125,453.88	95,771.77, 158,721.18
Maldives	26.49	21.86, 31.61	27.05	22.37, 32.28	29.29	24.05, 34.90	6.22	4.23, 8.68	620.39	510.83, 742.20
Burma	10,291.14	7,607.84, 14,071.02	10,613.63	7,896.38, 14,367.52	10,552.35	7,739.66, 14,580.53	2,328.96	1,471.64, 3,601.85	271,545.66	199,769.30, 374,582.84
Philippines	13,827.24	11,026.21, 17,100.02	13,964.25	11,341.93, 17,103.21	14,616.29	11,580.69, 18,202.20	3,186.74	2,119.98, 4,428.65	373,177.18	300,744.74, 458,874.13
Sri Lanka	2,506.52	1,822.45, 3,413.75	2,478.00	1,803.77, 3,369.01	2,833.98	2,039.40, 3,892.40	594.40	357.87, 901.65	61,124.63	44,055.48, 83,501.03
Thailand	22,545.27	17,018.46, 29,559.74	23,108.96	17,522.52, 30,147.58	24,360.83	18,211.19, 32,137.58	5,127.12	3,158.71, 7,512.38	524,356.39	389,890.72, 698,681.26
Vietnam	25,549.85	19,741.34, 32,387.02	25,160.99	19,493.53, 31,704.25	28,986.91	22,215.48, 37,389.29	5,927.12	3,923.01, 8,367.67	676,894.23	514,965.51, 873,764.92
High-income Asia Pacific										
Brunei	115.51	101.92, 130.45	103.45	91.60, 116.42	158.56	138.78, 179.78	27.44	19.35, 36.46	2,578.18	2,267.53, 2,923.29
Singapore	2,161.73	1,723.69, 2,713.17	1,565.88	1,413.73, 1,681.72	4,818.05	3,740.53, 6,167.55	609.71	414.16, 849.56	32,007.52	29,533.23, 34,270.58
North Africa and the Middle East										
Afghanistan	1,476.18	871.21, 2,335.11	1,492.27	891.59, 2,356.46	1,575.60	895.10, 2,505.23	345.82	177.19, 583.85	44,553.01	25,083.00, 71,319.35
Bahrain	140.72	106.64, 185.66	141.65	107.06, 186.90	154.53	116.66, 205.12	32.61	21.35, 48.65	3,546.09	2,674.88, 4,751.20
Egypt	6,123.00	4,303.05, 8,313.47	6,070.21	4,274.39, 8,216.10	6,731.47	4,752.21, 9,204.81	1,475.30	859.49, 2,283.70	174,974.92	123,445.87, 239,223.91
Iran	8,704.66	8,039.65, 9,366.32	8,923.24	8,247.20, 9,594.73	9,365.61	8,688.94, 10,053.26	2,023.34	1,445.19, 2,616.98	218,990.46	203,461.41, 234,522.71
Iraq	4,154.17	3,199.79, 5,128.84	4,231.66	3,274.77, 5,189.57	4,484.06	3,427.98, 5,628.77	957.44	629.52, 1,387.75	110,712.08	84,177.90, 139,886.99
Jordan	914.37	748.42, 1,109.93	917.40	749.29, 1,110.25	1,014.69	835.62, 1,232.58	216.36	142.14, 305.80	24,230.86	19,817.36, 29,412.55
Kuwait	225.33	184.58, 271.79	227.62	185.21, 274.59	258.87	215.66, 310.17	53.59	36.47, 74.87	5,540.99	4,539.37, 6,683.20
Lebanon	1,421.22	1,168.08, 1,867.91	1,433.09	1,184.23, 1,897.57	1,576.93	1,266.34, 2,051.06	319.65	213.78, 461.41	32,711.82	26,403.35, 42,585.37
Oman	146.86	116.66, 192.09	144.15	115.03, 187.19	168.86	132.47, 223.90	35.38	22.71, 52.66	3,875.69	3,001.88, 5,245.22
Palestine	523.49	443.92, 612.56	529.72	448.08, 617.82	568.27	481.15, 667.11	119.68	81.65, 162.68	14,203.36	12,042.86, 16,666.89
Qatar	124.76	88.77, 174.19	118.88	84.87, 165.47	152.93	107.22, 212.71	30.46	18.89, 47.90	3,497.10	2,442.30, 4,860.15
Saudi Arabia	1,544.89	1,187.06, 1,899.53	1,491.92	1,146.38, 1,829.70	1,848.16	1,411.21, 2,304.75	382.08	252.06, 553.89	45,487.57	34,469.24, 57,171.88
Syrian Arab Republic	1,372.09	1,006.81, 1,813.76	1,374.32	1,011.39, 1,813.03	1,509.37	1,100.36, 2,011.15	327.33	200.65, 485.27	36,951.19	26,937.04, 49,538.15
Turkey	29,510.56	23,370.09, 36,799.05	29,831.89	23,752.46, 37,028.28	31,739.56	25,030.55, 39,550.04	6,701.50	4,547.53, 9,477.92	743,637.07	585,408.24, 929,198.85
United Arab Emirates	541.68	393.37, 721.35	522.71	379.61, 696.70	627.40	453.55, 834.77	130.88	79.82, 199.43	16,697.74	12,066.29, 22,165.92
Yemen	1,302.23	885.33, 1,929.71	1,335.82	912.37, 1,971.91	1,345.21	909.07, 2,008.32	308.48	181.41, 504.79	36,208.29	24,430.35, 53,798.79
Central Europe										
Albania	1,174.38	861.50, 1,565.46	1,158.09	856.22, 1,531.85	1,330.78	964.24, 1,798.00	269.13	166.28, 405.31	25,926.45	18,893.57, 34,823.40
Bosnia and Herzegovina	2,434.60	1,889.96, 3,062.17	2,389.72	1,862.76, 2,986.22	2,753.69	2,126.68, 3,521.20	548.22	350.37, 785.91	56,744.93	43,565.61, 71,913.73
Bulgaria	4,837.61	3,859.19, 6,016.14	4,608.05	3,700.92, 5,714.36	5,737.34	4,490.47, 7,219.35	1,116.79	742.86, 1,571.90	116,517.16	91,572.63, 146,444.17
Croatia	3,430.52	2,706.26, 4,299.04	2,875.17	2,281.74, 3,607.40	5,337.58	4,123.26, 6,768.04	835.54	552.30, 1,173.56	64,967.90	50,856.24, 82,444.42
Czechia	6,942.77	5,695.06, 8,448.38	6,238.20	5,137.40, 7,580.23	9,367.69	7,573.79, 11,559.44	1,603.63	1,072.79, 2,221.38	133,507.49	108,873.62, 164,136.95
Hungary	9,509.80	7,849.06, 11,561.74	8,972.12	7,426.86, 10,848.33	11,679.77	9,517.11, 14,391.77	2,173.52	1,496.36, 2,962.28	212,473.60	173,458.78, 259,387.67
Montenegro	563.10	460.56, 685.03	531.01	437.42, 642.57	692.01	568.35, 840.10	129.94	85.64, 179.75	13,081.40	10,674.96, 15,950.14
Macedonia	1,339.70	1,025.41, 1,703.60	1,280.91	984.80, 1,625.14	1,598.14	1,208.28, 2,059.36	309.54	204.23, 443.17	33,072.32	25,235.20, 42,379.20
Poland	30,018.42	25,154.02, 35,717.87	31,205.87	26,089.58, 36,995.56	30,292.81	25,281.18, 36,146.49	6,569.69	4,446.70, 8,871.31	709,154.38	585,977.92, 846,722.76
Romania	11,544.70	9,482.85, 14,024.50	11,013.61	9,115.52, 13,362.10	13,733.60	11,257.44, 16,702.58	2,669.03	1,820.10, 3,667.51	273,221.57	223,948.07, 333,461.23
Serbia	7,699.56	6,057.60, 9,693.98	7,261.74	5,732.48, 9,070.69	9,489.67	7,405.09, 12,061.00	1,770.51	1,174.47, 2,490.33	176,690.62	137,757.74, 222,904.29
Slovakia	3,129.61	2,429.99, 4,052.56	2,529.50	1,975.84, 3,282.98	5,221.87	3,981.14, 6,781.43	787.63	508.69, 1,132.43	59,373.34	45,646.30, 77,497.77
Slovenia	1,395.08	1,081.70, 1,823.05	1,269.39	983.94, 1,644.08	1,980.98	1,511.20, 2,609.22	332.41	217.43, 468.18	27,659.86	21,218.09, 36,013.86
Eastern Europe										
Belarus	3,801.17	2,924.07, 4,935.88	3,543.49	2,747.82, 4,593.90	4,689.51	3,579.91, 6,124.14	889.54	574.52, 1,271.17	89,491.43	68,311.87, 117,562.44
Estonia	721.23	563.53, 905.64	714.05	560.79, 896.25	805.94	626.22, 1,015.01	163.17	107.96, 233.29	15,109.63	11,745.47, 19,172.87
Latvia	1,017.98	837.52, 1,237.70	950.20	787.73, 1,149.33	1,259.20	1,022.81, 1,549.30	236.21	161.52, 331.71	21,229.54	17,376.43, 25,943.39
Lithuania	1,395.18	1,139.09, 1,690.82	1,312.79	1,072.09, 1,586.10	1,673.09	1,352.39, 2,042.87	320.29	218.53, 443.07	29,561.11	23,885.14, 36,018.11
Moldova	1,067.45	919.87, 1,227.46	1,033.12	891.61, 1,184.85	1,218.09	1,046.71, 1,406.65	249.50	170.32, 348.20	27,405.49	23,509.08, 31,628.84
Russian Federation	58,183.52	49,720.66, 67,801.76	54,139.52	46,120.95, 63,100.09	74,012.48	63,149.49, 86,679.28	13,686.73	9,517.07, 18,088.91	1,345,629.42	1,140,036.53, 1,580,080.63
Ukraine	20,132.69	16,536.45, 24,383.32	17,023.08	14,127.24, 20,205.10	30,169.60	24,474.94, 36,910.48	5,007.00	3,353.86, 6,839.24	451,770.69	371,193.40, 538,851.86
Western Europe										
Cyprus	514.05	444.19, 591.86	461.97	403.78, 525.47	737.54	626.65, 856.86	124.12	88.25, 167.66	9,676.94	8,510.71, 10,948.28
Greece	9,237.89	7,274.26, 11,548.65	8,643.21	8,026.85, 9,193.05	12,189.65	9,411.58, 15,495.83	2,173.21	1,423.32, 3,070.77	172,150.80	161,828.94, 181,875.91
Israel	2,670.18	2,087.83, 3,391.41	2,518.13	2,313.63, 2,684.20	3,480.03	2,683.12, 4,483.52	633.62	413.76, 901.61	52,636.78	49,094.67, 55,907.37

YLDs, years lived with disability; DALYs, disability-adjusted life years; UI, uncertainty interval.

### The incidence, mortality, prevalence, YLDs, and DALYs in 1990 and 2019

**Figure 2:**
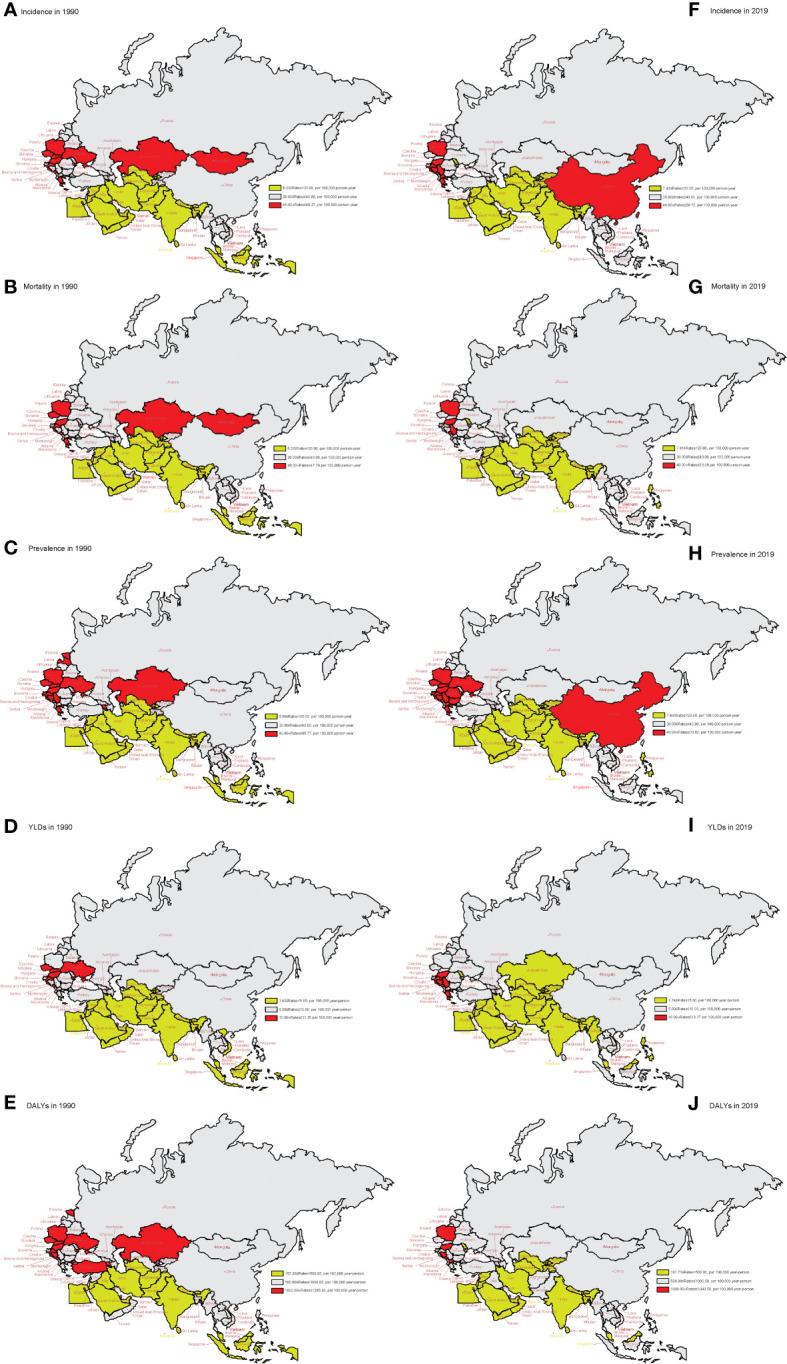
The age-standardized rates of incidence, mortality, prevalence, YLDs, and DALYs in 1990 and 2019 in “the Belt & Road” countries. **(A)** Age-standardized incidence rate in 1990. **(B)** Age-standardized mortality rate in 1990. **(C)** Age-standardized prevalence rate in 1990. **(D)** Age-standardized YLD rate in 1990. **(E)** Age-standardized DALY rate in 1990. **(F)** Age-standardized incidence rate in 2019. **(G)** Age-standardized mortality rate in 2019. **(H)** Age-standardized prevalence rate in 2019. **(I)** Age-standardized YLD rate in 2019. **(J)** Age-standardized DALY rate in 2019. YLDs, years lived with disability; DALYs, disability-adjusted life years.

**Figure 2** shows the age-standardized rates of incidence, mortality, prevalence, YLDs, and DALYs due to lung cancer in 1990 and 2019 in member countries of the “Belt and Road” Initiative. From 1990 to 2019, the incidence, mortality, prevalence, YLDs, and DALYs of lung cancer in South and Southeast Asia were generally low. In 1990, the country with the highest incidence of YLDs and DALYs of lung cancer was Hungary (49.27 per 100,000, 11.25 per 100,000, and 1,295.93 per 100,000). Bhutan had the lowest rates of incidence, mortality, prevalence, YLDs, and DALYs (6.03 per 100,000, 6.37 per 100,000, 5.90 per 100,000, 1.43 per 100,000, and 157.30 per 100,000, respectively). In 2019, Montenegro had the highest incidence, mortality, prevalence, YLDs, and DALYs (56.72 per 100,000, 53.36 per 100,000, 70.60 per 100,000, 13.17 per 100,000, and 1,343.58 per 100,000, respectively). Bangladesh had the lowest rates of incidence, mortality, prevalence, YLDs, and DALYs (7.43 per 100,000, 7.81 per 100,000, 7.40 per 100,000, 1.74 per 100,000, and 181.71 per 100,000, respectively). Prevalence, YLDs, and DALYs due to lung cancer declined most rapidly in Kazakhstan, while incidence, prevalence, and YLDs increased the fastest in China from 1990 to 2019. See **Supplementary Table 1** for more details.

### Trends in age-standardized incidence, prevalence, mortality, and DALYs

**Figure 3:**
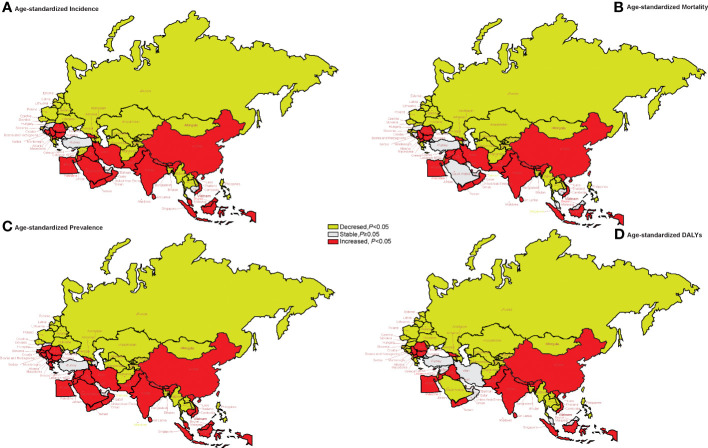
The trends of age-standardized rates of incidence, mortality, prevalence, and DALYs in 1990–2019 in “the Belt & Road” countries. **(A)** The AAPC of age-standardized incidence rate. **(B)** The AAPC of age-standardized mortality rate. **(C)** The AAPC of age-standardized prevalence rate. **(D)** The AAPC of age-standardized DALY rate. AAPC, average annual percent change; DALYs, disability-adjusted life years.

From 1990 to 2019, the AAPC of age-standardized incidence, prevalence, mortality, and DALYs generally showed a downward trend in Central Asia (except Georgia) and Eastern Europe, while in China, South Asia (except Bangladesh), and most countries in North Africa and the Middle East, the trend was mainly upward (**Figure 3**). The AAPC of age-standardized incidence, prevalence, mortality, and DALYs from lung cancer in China increased by 1.33% (95%CI: 1.15% to 1.50%, *p* < 0.001), 2.24% (95%CI: 2.10% to 2.38%, *p* < 0.001), 0.94% (95%CI: 0.74% to 1.14%, *p* < 0.001), and 0.42% (95%CI: 0.25% to 0.59%, *p* < 0.001), respectively. See **Supplementary Table 2** for more details.

### Trends in age-standardized YLDs

**Figure 4:**
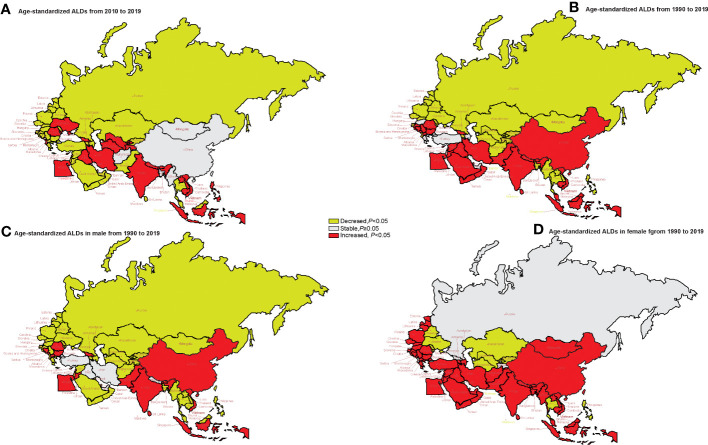
The trends of age-standardized rates of YLDs in genders in 2010–2019 and in 1990–2019 in “the Belt & Road” countries. **(A)** The AAPC of age-standardized rates of YLDs in 2010–2019. **(B)** The AAPC of age-standardized rates of YLDs in 1990–2019. **(C)** The AAPC of age-standardized rates of YLDs in men in 1990–2019. **(D)** The AAPC of age-standardized rates of YLDs in women in 1990–2019. AAPC, average annual percent change; YLDs, years lived with disability.

**Figure 4** shows the AAPC values of age-standardized YLD rate in member countries. Turkmenistan, Uzbekistan, Lao, the Philippines, Albania, and Ukraine had an upward trend of age-standardized YLDs from 2010 to 2019 and a downward trend from 1990 to 2019. Pakistan, Malaysia, Sri Lanka, Jordan, Oman, Qatar, Saudi Arabia, Syrian Arab Republic, Yemen, Bosnia and Herzegovina, Bulgaria, Macedonia, and Serbia showed a downward trend in age-standardized YLDs from 2010 to 2019, while an upward trend was observed from 1990 to 2019 (*p* < 0.05) (**Supplementary Table 3**). There were also differences in the trend of changes in AAPC between men and women from 1990 to 2019. A downward trend of the AAPC values of age-standardized YLD rate in men was shown in the vast majority of “B&R” countries. For women, the change trend of YLDs was stable in Georgia and Russia, while the upward trend was observed in most other countries (**Supplementary Table 4**).

### Trends in age-standardized YLDs stratified by age groups

**Figure 5:**
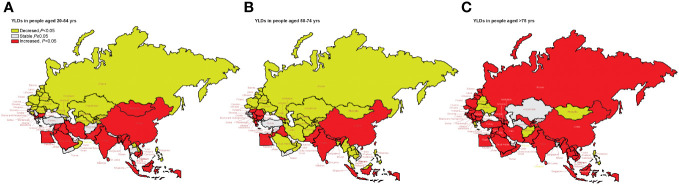
Visualization of the trends of age-standardized YLD rate stratified by age from 1990 to 2019 in “the Belt & Road” countries. **(A)** YLD rate in people aged 20–54 years. **(B)** YLD rate in people aged 55–74 years. **(C)** YLD rate in people aged ≥75 years. YLDs, years lived with disability.

**Figure 5** shows the long-term trends of age-standardized YLD rate due to lung cancer, stratified by age from 1990 to 2019 for the “B&R” countries. We found that in Maldives, the Philippines, Bahrain, Belarus, and Ukraine, the age-standardized YLDs of all ages showed a downward trend, while in China, Bhutan, India, Pakistan, Indonesia, Malaysia, Sri Lanka, Egypt, Iraq, Jordan, Lebanon, Bulgaria, Montenegro, Macedonia, Serbia, and Cyprus, the age-standardized YLDs of all ages showed an upward trend (*p* < 0.05). For adults aged 75 years or older, the age-standardized YLD rate from 1990 to 2019 showed an increasing trend in the “B&R” countries, except Kazakhstan, Kyrgyzstan, Turkmenistan, Mongolia, Bangladesh, Maldives, Afghanistan, Bahrain, the United Arab Emirates, Belarus, Moldova, Ukraine, Greece, and the Philippines. In China, age-standardized YLDs showed an increasing trend with the increase of age, and the highest AAPC value of age-standardized YLD rate from 1990 to 2019 was in adults aged 75 years or older: 2.87% (95%CI: 2.60%–3.14%, *p* < 0.001). See **Supplementary Table 5** for more details.

### Trends in age-standardized DALYs stratified by risk factors

**Figure 6:**
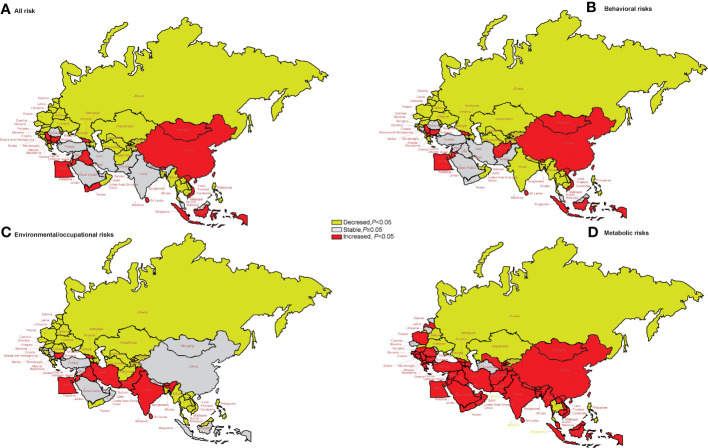
The temporal trend in the DALY rate of lung cancer attributed to risk factors for 1990–2019 in the “B&R” countries. **(A)** All risk factors. **(B)** Behavioral risks. **(C)** Environmental/occupational risks. **(D)** Metabolic risks. DALYs, disability-adjusted life years.

**Figure 6** shows the long-term trends of the age-standardized DALY rate due to lung cancer, stratified by risk factors from 1990 to 2019 for the “B&R” countries. We found that in middle SDI regions, China, Georgia, Bhutan, Indonesia, Sri Lanka, Vietnam, Egypt, Iraq, Jordan, Lebanon, Palestine, Yemen, Bulgaria, Montenegro, Macedonia, Serbia, and Cyprus, the age-standardized DALYs due to all risk factors showed an upward trend, while globally and in the other “B&R” countries, the age-standardized DALYs of all risk factors showed a downward trend (*p* < 0.05).

For DALYs of lung cancer attributable to behavioral risks, the age-standardized DALY rate of middle SDI regions, China, Georgia, Bhutan, Bhutan, Indonesia, Sri Lanka, Vietnam, Afghanistan, Egypt, Jordan, Lebanon, Palestine, Bulgaria, Montenegro, Macedonia, Serbia, and Cyprus showed an increasing trend in the “B&R” countries from 1990 to 2019 (all *p* < 0.05).

For DALYs of lung cancer due to environmental/occupational risks, the age-standardized DALY rate of Georgia, Bhutan, Pakistan, Sri Lanka, Egypt, Iran, Iraq, Jordan, Lebanon, and Bulgaria showed an increasing trend in the “B&R” countries from 1990 to 2019 (all *p* < 0.05).

For DALYs of lung cancer attributable to metabolic risks, the age-standardized DALY rate of Kazakhstan, Kyrgyzstan, Turkmenistan, Maldives, the Philippines, Thailand, Singapore, Bahrain, Slovakia, Belarus, and Ukraine showed a decreasing trend in the “B&R” countries from 1990 to 2019 (all *p* < 0.05). See **Supplementary Table 6** for more details.

## Discussion

With an estimated 1.79 million deaths per year, lung cancer is one of the leading causes of cancer-related deaths (5). Smoking, poor diet, lack of exercise, genetic factors, air pollution, and occupational exposure are all risk factors for cancer (13). Smoking is an important risk factor for increasing cancer risk (14). Cigarettes contain polycyclic aromatic hydrocarbons and nitrosamines. Nicotine is an addictive substance, so it leads to frequent use among smokers, and therefore, lung cancer is more common among them (15, 16). In the last decade, the age-standardized incidence rate in high-socio-demographic index countries has been decreasing due to tobacco control (17). We found that the incidence, prevalence, and YLDs increased the fastest in China from 1990 to 2019, and the age-standardized incidence, prevalence, mortality, and DALYs showed an upward trend in China, South Asia, North Africa, and the Middle East, which may be related to a large number of smokers in these countries.

Our study found significant differences in the trend of age-standardized YLDs between genders. A downward trend of the AAPC values of age-standardized YLD rate in men was shown in the “B&R” countries. For women, the upward change trend of YLDs was observed in most countries. The global incidence of lung cancer in men is declining twice as fast as in women (5). The age-standardized incidence rates of lung cancer among women are predicted to increase before 2035 and are expected to peak after the 2020s, while those among men are expected to decrease in almost all countries (18). The mortality of cancers due to smoking has substantially increased among women in most countries of the North Africa and Middle East region (19). These studies all suggest that the “B&R” and even countries around the world need to strengthen the publicity and education of female smoking cessation and attach importance to physical examination and lung screening, which will help control the incidence rate and mortality of female lung cancer.

The increase in life expectancy has led to a greater global burden of diseases. Global population aging is the principal medical and social demographic problem worldwide. In the Non-Organisation for Economic Co-operation and Development countries, the fastest-aging countries are Saudi Arabia, Brazil, and China (20). Since 2000, China has gradually entered an aging society, the aging in China has not been alleviated but has gradually increased recently, and the burden of lung cancer on elderly patients is also increasing (21). In the “B&R” member countries, the age-standardized YLDs in most countries showed an upward trend with the increase of age, and the highest AAPC value of the age-standardized YLDs in 1990–2019 was in adults aged 75 years or older. A satisfactory and appropriate understanding of the health problems of older people caused by aging is a common challenge in the world. The goal vision is to establish a world where everyone has the chance to live a healthy and long life (20). This requires close cooperation between multiple sectors and departments in the “B&R” member countries to promote healthy aging.

In recent decades, countries within the Middle East have faced social, political, and financial instability brought about by war. These conflicts have directly led to a significant decline in the overall level of local medical services and a shortage of professional experts, seriously affecting the provision of cancer diagnosis services. The cancer patients in these areas cannot be diagnosed early and cannot receive effective healthcare (22, 23). In addition, the use of depleted uranium and white phosphorus bombs in wars may cause environmental pollution and even cancer (24). Therefore, many cancer patients must bear the cost of traveling to neighboring countries in order to receive medical services. Our study also found that from 1990 to 2019, the AAPC of age-standardized incidence rate, morbidity, mortality, and DALYs showed an upward trend in most countries in the Middle East. It is important to alleviate the shortage of medical services for these countries through the “B&R” Initiative.

With a deeper understanding of the biology of lung cancer, many advances have been made in the treatment of lung cancer, such as minimally invasive techniques, stereotactic ablative radiotherapy, targeted therapies, and ICIs (25). New therapies have benefited patients and reduced the burden of disease. However, due to various reasons such as economic development and healthcare systems, countries have varying opportunities to access drugs and healthcare (5). In low-income countries, new lung cancer cases and mortality continue to increase, which may be related to limited access to healthcare and outdated treatment methods in these countries (5). By implementing large-scale infrastructure construction and trade facilitation, poor and low-income countries can return to the mainstream of global development from a state of global marginalization, thereby providing bright prospects for comprehensive and long-term economic growth in the “B&R” member countries. In addition, the medical field should also be highly valued. The exchange of medical knowledge and experience among medical institutions in the “B&R” countries should be continuously promoted so that medical technology and health services will be extended from higher-level countries to lower-level ones, thus improving the medical level of each country and benefiting low-income people.

YLDs can reflect the amount of time lived in states of less than good health due to a specific disease or injury and are calculated as the prevalence of a sequela of any given cause multiplied by the average duration until death or remission and by the disability weight for that sequela. The YLDs are the sum of each of the sequelae associated with the disease or injury (26, 27). YLL refers to the loss of life caused by early death. Although YLDs and YLLs can reflect the burden on society, YLDs are more likely to be affected by diseases and injuries in their lives. Reducing the burden of disease involves not only prolonging the survival period of patients but also improving the quality of life of patients. The interventions required to reduce the causes of death may differ from those needed to reduce risk factors and disability rates for disease burden. This is why we chose to calculate YLDs in this study.

Globally, from 2010 to 2019, the number of lung cancer increased by 23.3%, and the age-standardized incidence rates decreased by 7.4% in men and increased by 0.9% in women (4). Compared to the USA and UK, China had lower incidence but higher cancer mortality and DALYs (28). All the age-standardized incidences had a decreasing trend in men and an increasing trend in women from 1990 to 2019 in the North Africa and Middle East region. Over 80% of DALYs could be decreased by controlling tobacco use (23). The number of new cases is predicted to increase by 50.19% from 2010 to 2035. When stratified by geographic region, the most rapid increases were predicted in Eastern Asia (79.00% for men and 140.05% for women) (18). We found that in the “B&R” countries, especially in middle SDI regions, DALYs due to all risk factors showed an upward trend, while globally, DALYs had a downward trend.

Unlike previous lung cancer burden studies based on GBD data, this study focuses on the “B&R” countries proposed by China, the world’s second-largest economy, under the global community of shared future strategy. It not only describes the changes in disease burden in a specific region or globally but also provides targeted data support for how countries with significant differences in social demographic indices but strong political and economic connections can formulate policies to reduce the burden of lung cancer. Preventive measures such as smoking control interventions and air quality management should be prioritized in low and middle SDI regions. Our research also suggested that we should pay more attention to female lung cancer patients. For women, the upward trend of YLDs was observed in the “B&R” countries, and it may continue to rise in the future (18). By studying the continuous transformation of epidemiology in the “B&R” countries, the necessity of resource redistribution and improvement of lung cancer control measures is highlighted.

This study also has several limitations. First, GBD 2019 has inherent limitations that are applicable to this study. Second, the GBD database lacks lung cancer’s pathological staging and classification. In the future, the “B&R” countries can use economic development as a link to drive the construction of information-based disease monitoring systems, providing sufficient support for the estimation of disease burden and policy adjustments.

## Conclusion

In summary, the overall burden of lung cancer in the “B&R” countries is still huge, especially in China, South Asia, North Africa, and the Middle East. There are significant differences between genders and ages. The lung cancer prevention and treatment policies in women and adults aged 75 years or older need to be improved. With the background of the health “B&R” Initiative, multi-country cooperation and experience sharing will play an important role in jointly facing the challenges caused by lung cancer and promoting the positive development of healthcare in all member countries.

## Data availability statement

The original contributions presented in the study are included in the article/**Supplementary Material**. Further inquiries can be directed to the corresponding author.

## Author contributions

XL conceived and designed the study. ZZhu, WY, LZ, WJ, BC,QW, XC, SY, and ZZhang analyzed the data. ZZhu, XL, and YD provided significant advice and consultation. WY and XL wrote the manuscript. All authors contributed to the article and approved the submitted version.

## Supplementary material

Supplementary Table 1 The age-standardized rates of incidence, mortality, prevanlence, YLDs and DALYs in member countries of the “Belt and Road” initiative from 1990 to 2019. (YLDs, years lived with disability; DALYs, disability-adjusted life-years; UI, uncertainty interval).

Supplementary Table 2 The average annual percentage change (AAPC) of age-standardized incidence, mortality, prevanlence and DALYs from 1990 to 2019 in the “Belt & Road” countries. (AAPC, average annual percent change; DALYs, disability-adjusted life-years).

Supplementary Table 3 The average annual percentage change (AAPC) of age-standardized YLDs in 2010-2019 and 1990-2019 in the “Belt & Road” countries. (AAPC, average annual percent change; YLDs, years lived with disability).

Supplementary Table 4 The average annual percentage change (AAPC) of age-standardized rates for YLDs in male and female from 1990 to 2019 in the “Belt & Road” countries. (AAPC,average annual percent change; YLDs, years lived with disability).

Supplementary Table 5 The average annual percentage change (AAPC) of age-standardized rates for YLDs stratified by age from 1990 to 2019 in the “Belt & Road” countries. (AAPC,average annual percent change; YLDs, years lived with disability).

Supplementary Table 6 The average annual percentage change (AAPC) of age-standardized rates for DALYs stratified by risk factors from 1990 to 2019 in the “Belt & Road” countries. (AAPC, average annual percent change; DALYs, disability-adjusted life-years).
