# Supplementary material for: Diversities of disability caused by lung cancer in the 66 Belt and Road initiative countries: a secondary analysis from the Global Burden of Disease Study 2019
Source: Front Oncol. 2023 Nov 10;13:1247006. doi: 10.3389/fonc.2023.1247006 (PMC10668146; doi:10.3389/fonc.2023.1247006)
Supplement: Supplementary Table 1 — The age-standardized rates of incidence, mortality, prevanlence, YLDs and DALYs in member countries of the “Belt and Road” initiative from 1990 to 2019. (YLDs, years lived with disability; DALYs, disability-adjusted life-years; UI, uncertainty interval). [file Table_1.docx]

**GATHER checklist of information included in reports of global health estimates**

| **#** | **Checklist item** | **Section/paragraph/** **interpretation** |
| --- | --- | --- |
| **Objectives and funding** | | |
| 1 | Define the indicators, populations, and time periods for which estimates were made. | Methods / “Data Sources” |
| 2 | List the funding sources for the work. | Funding |
| **Data Inputs** | | |
| *For all data inputs from multiple sources that are synthesized as part of the study:* | | |
| 3 | Describe how the data were identified and how the data were accessed. | As mentioned in the Methods / “Data Sources” section, the details have been published previously. |
| 4 | Specify the inclusion and exclusion criteria. Identify all ad-hoc exclusions. | As mentioned in the Methods / “Data Sources” section, the details have been published previously. |
| 5 | Provide information on all included data sources and their main characteristics. For each data source used, report reference information or contact name/institution, population represented, data collection method, year(s) of data collection, sex and age range, diagnostic criteria or measurement method, and sample size, as relevant. | Available via online data source tools (http://ghdx.healthdata.org/gbd-2019/data-input-sources). |
| 6 | Identify and describe any categories of input data that have potentially important biases (e.g., based on characteristics listed in item 5). | As mentioned in the Methods, the details have been published previously. |
| *For data inputs that contribute to the analysis but were not synthesized as part of the study:* | | |
| 7 | Describe and give sources for any other data inputs. | Available via online data source tools (http://ghdx.healthdata.org/gbd-2019/data-input-sources). |
| *For all data inputs:* | | |
| 8 | Provide all data inputs in a file format from which data can be efficiently extracted (e.g., a spreadsheet as opposed to a PDF), including all relevant meta-data listed in item 5. For any data inputs that cannot be shared due to ethical or legal reasons, such as third-party ownership, provide a contact name or the name of the institution that retains the right to the data. | Available via online data source tools (http://ghdx.healthdata.org/gbd-2019/data-input-sources) |
| **Data analysis** | | |
| 9 | Provide a conceptual overview of the data analysis method. A diagram may be helpful. | Flow diagrams of the overall methodological processes were available online  (http://ghdx.healthdata.org/gbd-2019/code/nonfatal-12) |
| 10 | Provide a detailed description of all steps of the analysis, including mathematical formulae. This description should cover, as relevant, data cleaning, data pre-processing, data adjustments and weighting of data sources, and mathematical or statistical model(s). | As mentioned in the Methods / “Statistical Analysis” section, |
| 11 | Describe how candidate models were evaluated and how the final model(s) were selected. | As mentioned in the Methods / “Statistical Analysis” section, the details have been published previously. |
| 12 | Provide the results of an evaluation of model performance, if done, as well as the results of any relevant sensitivity analysis. | As mentioned in the Methods / “Statistical Analysis” section, the details have been published previously. |
| 13 | Describe methods for calculating uncertainty of the estimates. State which sources of uncertainty were, and were not, accounted for in the uncertainty analysis. | Methods / “Statistical Analysis” section |
| 14 | State how analytic or statistical source code used to generate estimates can be accessed. | Methods / “Statistical Analysis” section |
| **Results and Discussion** | | |
| 15 | Provide published estimates in a file format from which data can be efficiently extracted. | Results, and online data tools (data visualization tools, and data query tools,  http://ghdx.healthdata.org/gbd-2019) |
| 16 | Report a quantitative measure of the uncertainty of the estimates (e.g. uncertainty intervals). | Results, and online data tools (data visualization tools, and data query tools,  http://ghdx.healthdata.org/gbd-2019) |
| 17 | Interpret results in light of existing evidence. If updating a previous set of estimates, describe the reasons for changes in estimates. | Discussion, paragraphs 1-7 |
| 18 | Discuss limitations of the estimates. Include a discussion of any modelling assumptions or data limitations that affect interpretation of the estimates. | Discussion, paragraph 8 |
